# Botanical interventions for alcohol hangover: a scoping review

**DOI:** 10.3389/fphar.2026.1945900

**Published:** 2026-10-26

**Authors:** Xiaodan Wang, Meijin Song, Lei Zhou, Junjie Yao, Yingzi Cui, Jiajuan Guo, Tianying Chang, Xing Liao

**Affiliations:** 1 Institute of Basic Research in Clinical Medicine, China Academy of Chinese Medical Sciences, Beijing, China; 2 College of Chinese Medicine, Changchun University of Chinese Medicine, Changchun, China; 3 Department of Pathology, First Affiliated Hospital to Changchun University of Chinese Medicine, Changchun, China; 4 Guang’anmen Hospital, China Academy of Chinese Medical Sciences, Beijing, China; 5 Evidence-Based Medicine Office, First Affiliated Hospital to Changchun University of Chinese Medicine, Changchun, China; 6 College of Traditional Chinese Medicine, Changchun University of Chinese Medicine, Changchun, China

**Keywords:** alcohol hangover, botanical interventions, phytochemical characterization, plant extracts, scoping review

## Abstract

**Background:**

Botanical interventions are widely studied and marketed for alcohol hangover, but direct symptom evidence, biomarker findings, and preclinical results are frequently conflated. This scoping review mapped the clinical and preclinical evidence for botanical interventions for alcohol hangover and examined the reproducibility of botanical and extract reporting.

**Methods:**

PubMed, Embase, Web of Science, the Cochrane Library, CBM, CNKI, VIP, and Wanfang Data were searched from inception to 22 July 2025. Citation searching and peer-reviewer recommendation were used as additional identification methods. Evidence was organized as Level 1 (direct human hangover symptoms), Level 2 (human pharmacokinetic/exposure, biochemical, mechanistic, and pharmacogenetic outcomes), and Level 3 (preclinical animal evidence); safety was charted separately. Botanical identity and extract characterization were charted using a ConPhyMP-informed framework.

**Results:**

Twenty-two studies reported in 21 publications were included: 15 human studies and seven animal studies, with one publication containing both human and animal experiments. Across the human studies, 470 participants were enrolled or randomized and 437 were included in the reported analyses. Sex was available for 400 analyzed participants, of whom 293 (73.3%) were male. Direct symptom evidence was heterogeneous: seven publications showed a positive signal, six were mixed, and two were null. Pharmacokinetic or biomarker changes were not treated as substitutes for symptom improvement. Scientific botanical identity was reported in 19/21 publications, whereas authentication and voucher numbers were reported in only 3/21 each; quantitative marker analysis or a defined phytochemical profile was reported in 8/21. Safety reporting was inconsistent.

**Conclusion:**

Several botanical interventions showed preliminary signals in selected hangover symptoms or alcohol-related biomarkers, but the clinical evidence remains limited and heterogeneous and includes mixed and null findings. The current evidence is insufficient to establish routine therapeutic efficacy or long-term safety. Future studies should prioritize adequately powered trials with validated symptom outcomes, clear prevention-versus-treatment timing, and reproducible botanical and phytochemical characterization.

**Systematic Review Registration:**

https://osf.io/cv83d, identifier https://doi.org/10.17605/OSF.IO/CV83D.

## 1 Introduction

Alcohol hangover is a transient constellation of negative physical and mental symptoms that develops after a single episode of alcohol consumption, typically as blood alcohol concentration approaches or returns to zero (Verster, 2008; Verster et al., 2010). Common manifestations include headache, fatigue, nausea, thirst, dizziness, mood disturbance, and impaired concentration. Hangover can impair work performance and driving-related function and contributes to the broader social burden associated with alcohol consumption (Frone, 2006; Høiseth et al., 2015).

Alcohol hangover should be distinguished from alcohol use disorder (AUD). Hangover is an acute post-intoxication syndrome, whereas AUD is a chronic disorder characterized by impaired control over alcohol use and clinically significant consequences. Pharmacotherapies used in AUD management, including naltrexone, acamprosate, and disulfiram, are intended to reduce drinking, craving, or relapse rather than to treat the transient symptom complex of hangover (Patel and Balasanova, 2021). The World Health Organization Global alcohol action plan 2022-2030 emphasizes evidence-based strategies to reduce alcohol-related harm at population and health-system levels (World Health Organization, 2024). Research on hangover interventions should therefore be viewed as addressing an immediate alcohol-related outcome, not as a substitute for prevention or treatment of harmful alcohol use or AUD.

No medicine is specifically approved as a definitive treatment for alcohol hangover. The U.S. Food and Drug Administration has taken action against products marketed with unsubstantiated hangover claims (Voelker, 2020), and marketed hangover products frequently lack direct peer-reviewed evidence for efficacy and safety (Verster et al., 2021). Botanical preparations are nevertheless widely investigated because they may contain multiple plant-derived metabolites with effects on ethanol or acetaldehyde exposure, inflammatory signaling, oxidative stress, gastrointestinal symptoms, or other pathways proposed to contribute to hangover (Palmer et al., 2019). These mechanistic hypotheses, however, should not be equated with clinical symptom benefit.

The evidence base is also methodologically uncertain. A 2022 systematic review of placebo-controlled randomized trials found that efficacy estimates for pharmacologically active hangover interventions were of very low quality, largely because of methodological limitations and imprecision (Roberts et al., 2022). Since that review, additional botanical trials have appeared, including reports published in 2023–2024 and the FHR-SM trial first published online in 2025 before assignment to a 2026 issue (Jung et al., 2023; Lee et al., 2024; Paik et al., 2024; Hong et al., 2026). At the same time, botanical research raises a distinct reproducibility problem: the biological activity of an extract depends on plant identity, plant part, processing, extraction, and chemical characterization. The ConPhyMP guidelines provide a framework for reporting these features in pharmacological and clinical research using complex extracts (Heinrich et al., 2022).

Accordingly, this scoping review aimed to map the range and characteristics of botanical interventions studied for alcohol hangover; distinguish direct human symptom evidence from human pharmacokinetic/biochemical outcomes and preclinical animal evidence; describe intervention timing, safety reporting, and pharmacogenetic findings; and assess the completeness of botanical identification and extract characterization using a ConPhyMP-informed data-charting framework.

## 2 Methods

This scoping review was conducted using the Joanna Briggs Institute approach to scoping reviews and reported in accordance with PRISMA-ScR (Tricco et al., 2018). The review was registered on the Open Science Framework (DOI: https://doi.org/10.17605/OSF.IO/CV83D). The revised manuscript does not characterize the registration as prospective because the temporal relationship between the registration and all review activities could not be verified from the materials available for this revision. Clarifications introduced during revision included explicit evidence-level classification, a more restrictive distinction between hangover and acute intoxication or organ injury, and separate reporting of records identified through methods other than the original electronic database search.

### 2.1 Information sources and search strategy

We systematically searched PubMed, Embase, the Cochrane Library, Web of Science, CBM, CNKI, VIP, and Wanfang Data from database inception to 22 July 2025. The complete search strategies are provided in Supplementary File S1. In addition, reference lists of relevant reviews were citation searched, and records identified through peer-reviewer recommendation were evaluated using the same eligibility criteria. These additional records are reported separately under “other methods” in the PRISMA flow diagram rather than as an electronic database update.

The original electronic search identified 5,225 records: PubMed (n = 712), Embase (n = 1,087), Web of Science (n = 721), Cochrane Library (n = 158), CNKI (n = 163), Wanfang Data (n = 1,430), VIP (n = 113), and CBM (n = 841). The four Chinese databases were retained to reduce database and language bias in a topic involving traditional and botanical preparations. No Chinese-language primary report met the final eligibility criteria.

### 2.2 Eligibility criteria

Eligibility was defined using the Population-Concept-Context framework.

#### 2.2.1 Population

Human studies were eligible when participants underwent an alcohol challenge or were assessed during an established post-alcohol hangover state and the study evaluated direct hangover symptoms and/or hangover-related human outcomes. Animal studies were eligible when the report explicitly framed the *in vivo* experiment as investigating hangover or post-alcohol effects and included at least one predefined behavioral or biological alcohol-exposure endpoint plausibly relevant to that aim. Because animals cannot report subjective hangover, animal studies were not treated as direct clinical symptom evidence.

#### 2.2.2 Concept

Eligible interventions were botanically derived preparations used in relation to alcohol hangover, including single botanical drugs or extracts, multi-botanical formulations, fresh juices or other botanical preparations, and standardized or commercial botanical preparations. The original botanical identity reported by study authors was retained separately from review-level taxonomic normalization. Chemically isolated plant-derived metabolites were not treated as equivalent to complex botanical preparations and were excluded when the study context was acute intoxication rather than hangover.

#### 2.2.3 Context and study types

There was no restriction on study setting or participant demographic characteristics, but eligible reports were limited to English or Chinese. Human randomized or non-randomized controlled studies, crossover studies, self-controlled studies, and prospective observational/registry studies were eligible. Animal *in vivo* controlled experiments meeting the hangover-directed criterion above were eligible. Systematic reviews, meta-analyses, editorials, and commentaries were not included as evidence sources; their reference lists could be used for citation searching.

#### 2.2.4 Exclusion criteria

Studies were excluded when they addressed acute alcohol intoxication or drunkenness without an eligible post-alcohol hangover outcome; alcohol poisoning; chronic alcohol-related organ injury or AUD; organ protection without a hangover-directed aim; molecular or *in vitro* experiments without an eligible human or *in vivo* evidence unit; a non-botanical intervention outside the prespecified concept; duplicate reports; or an ineligible publication type. For full-text exclusions, one principal reason was assigned to each report. Loss or recovery of the righting reflex was classified as an acute central nervous system sedation/recovery measure and not as a validated surrogate for subjective human hangover.

### 2.3 Study selection and data charting

Records were managed in EndNote X9. Two reviewers independently screened titles/abstracts and full texts; disagreements were resolved by discussion and, when necessary, by a third reviewer. Data were charted at the publication level and linked to a trial-level identifier when multiple reports could potentially arise from the same participant cohort. Potential overlap between the two 2024 Hovenia trials was specifically assessed using trial registrations, ethics identifiers, recruitment information, and study setting.

For human studies, we charted study design and blinding, randomized/enrolled and analyzed sample sizes, participant sex and age, alcohol challenge, intervention dose and administration timing, timing of symptom, pharmacokinetic, biochemical, and other outcome assessments, direct symptom instruments, outcome roles assigned by the original authors (primary, secondary, exploratory, or not specified), pharmacokinetic and biochemical outcomes, statistical direction, and safety reporting. For animal studies, we additionally charted species/strain, alcohol-exposure model, intervention timing, outcome-assessment time points, behavioral versus metabolic/biochemical endpoints, organ-injury outcomes, and the interpretive relevance of the model to human hangover.

### 2.4 Evidence hierarchy and synthesis

Evidence was organized into three interpretive levels. Level 1 comprised direct human hangover symptom outcomes. Level 2 comprised human pharmacokinetic/exposure, biochemical, mechanistic, and pharmacogenetic outcomes. Level 3 comprised eligible preclinical animal evidence. Safety was charted and reported separately rather than treated as an efficacy evidence level. Only Level 1 findings were used to characterize direct symptom change; Level 2 and Level 3 findings were used as supportive or mechanistic context. Positive, mixed, and null labels were descriptive mappings of direct human symptom findings rather than quality or certainty ratings.

Consistent with the purpose of a scoping review, no formal risk-of-bias or certainty-of-evidence assessment was performed. Study-design characteristics were charted and reported for transparency, and limitations arising from uncontrolled designs, small samples, heterogeneous interventions, and outcome selection were incorporated into the interpretation.

### 2.5 Botanical identification and ConPhyMP-informed extraction

Botanical identity, material source, plant part, authentication, voucher documentation, extraction solvent and procedure, drug-to-extract ratio (DER) or extraction yield, phytochemical characterization, marker compounds, analytical method or fingerprint information, manufacturer, and batch/lot information were charted using a ConPhyMP-informed framework (Heinrich et al., 2022). The botanical name reported in each primary article was retained separately from the accepted botanical name normalized by the review team using authoritative botanical databases. Because this was a scoping review rather than a primary extract-characterization study, ConPhyMP was applied as a review-level reporting-audit framework and did not imply that every included primary study fulfilled each ConPhyMP criterion. Information absent from the primary publication was recorded as not reported (NR) and was not inferred. DER or yield values calculated by the review team from explicit source masses were labelled as review-calculated rather than author-reported.

## 3 Results

### 3.1 Study selection and evidence set

The database search identified 5,225 records. After removal of 902 duplicates, 4,323 records were screened; 4,166 were excluded at title/abstract screening and 157 reports were assessed in full text. Of these, 141 were excluded, leaving 16 eligible reports from the database-search branch. Citation searching and peer-reviewer recommendation identified five additional reports; all five were retrieved, assessed, and included. In total, 22 studies reported in 21 publications were included in the scoping review (Figure 1): 15 human studies and seven animal studies, with one Noni publication containing both human and animal experiments and therefore contributing to both evidence strata while counting once at the publication/report level. The individual studies are summarized in Tables 1–3.

**FIGURE 1:**
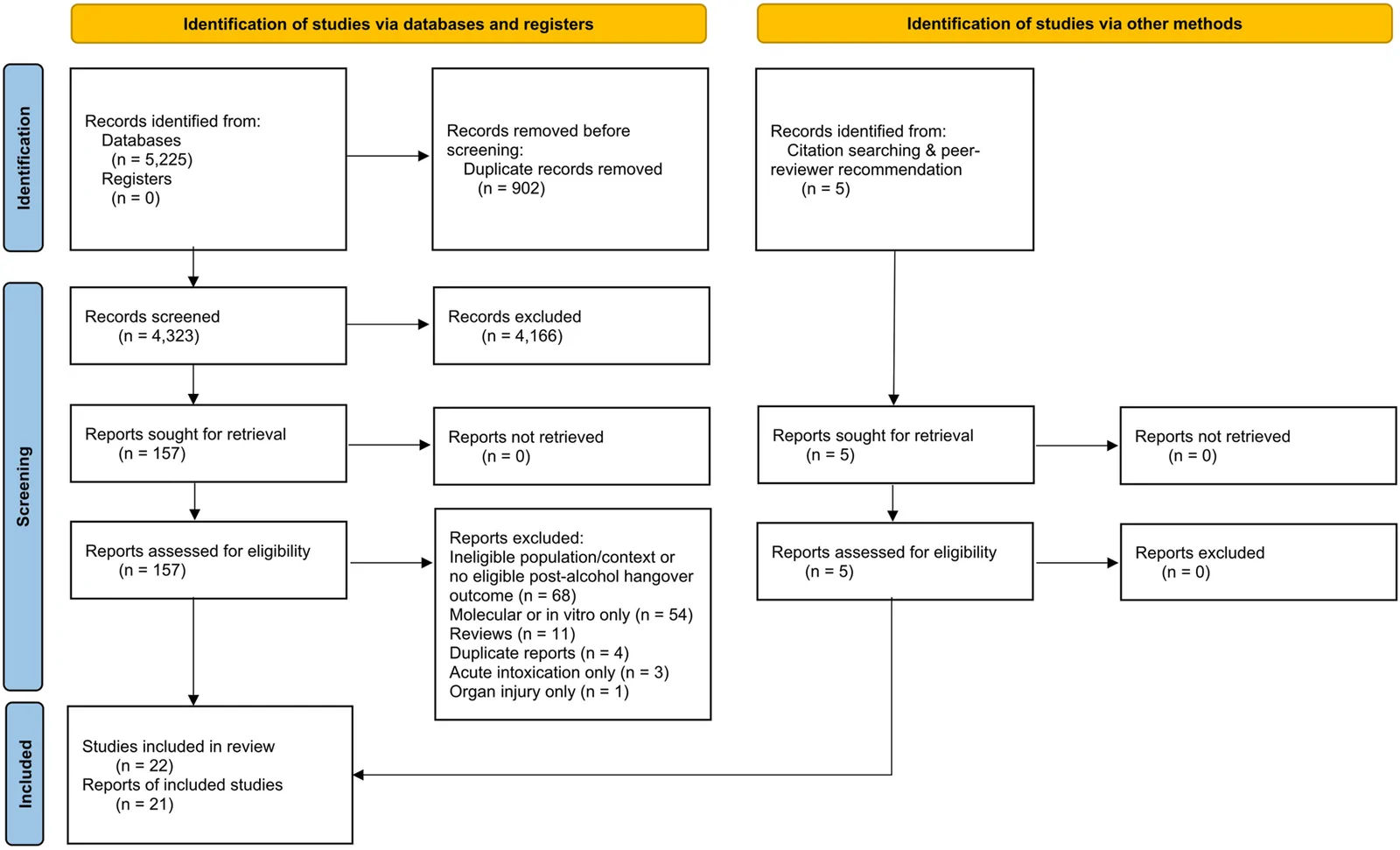
PRISMA 2020 flow diagram for new systematic reviews which included searches of databases, registers and other sources.

**TABLE 1:** Characteristics of included human intervention studies.

Study	Design	Participants	Alcohol challenge	Botanical intervention/reported identity	Preparation/dose	Administration timing	Outcome assessment time point(s)
P01 Pittler et al., 2003 (UK)	Randomized, double-blind, placebo-controlled crossover	15 analyzed; 5 M/10 F; age reported by sequence: 42.0 ± 12.8 vs. 40.6 ± 10.2 years	Self-selected beverages; ∼1.2 g alcohol/kg	Standardized artichoke extract LI120; reported *Cynara scolymus*; plant part NR	3 × 320 mg immediately before +3 × 320 mg immediately after alcohol (1.92 g/day)	Pre + post alcohol	Primary hangover VAS: 10 h after alcohol exposure (09:00); mood/cognition: 1 h pre-alcohol and 10 h post-alcohol; BP/HR: 1 h pre-alcohol and following morning; symptom resolution tracked for 24–48 h
P02 Wiese et al., 2004 (USA)	Randomized, double-blind, placebo-controlled crossover	64 randomized; 55 completed; 23 M/32 F; age 21–35 years	Vodka/gin/rum/bourbon/scotch/tequila; up to 1.75 g/kg over 4 h	*Opuntia ficus-indica* fruit extract (Tex-OE)	1600 IU (2 × 800 IU capsules)	5 h before alcohol	Baseline measures; following morning on return to the study facility: Blood/urine sampling, BAC, and hangover symptom assessment (exact hours after drinking completion NR)
P03 Takahashi et al., 2010 (Japan)	Exploratory repeated self-controlled testing; no randomized placebo control	10; 4 M/6 F; 36.9 ± 13.0 years	Alcohol type NR; >2 units (∼44 g pure alcohol)	KSS: pith of *Citrus tangerine* + rhizome of *Zingiber officinale* + brown sugar	Daily botanical equivalent 3 g Kitsuraku +6 g Shokyo; aqueous decoction/granules	Test-specific: Post-alcohol; repeated pre + post; or pre-alcohol	Symptoms/VAS: Four post-alcohol assessments - next morning, next evening, morning of the day after next, and evening of the day after next; time to complete symptom resolution also recorded
P04 Lee et al., 2013 (Korea)	Randomized, single-blind crossover	20 enrolled/selected; 14 completed/analyzed; 14 M; 25.8 ± 0.86 years	Soju 20.1%, 540 mL	Korean pear juice; *Pyrus pyrifolia* fruit	220 mL fresh juice	30 min before alcohol	Hangover questionnaire: 1, 4, and 15 h; blood alcohol/acetaldehyde and hematology: Pre-drinking/baseline and 0, 0.25, 0.5, 1, 2, 4, 6, and 15 h
P05 Lee et al., 2014 red ginseng (Korea)	Randomized crossover; water control	25; 25 M; 28.1 ± 0.6 years	Scotch whisky 40%, 100 mL	Red ginseng extract beverage; scientific species/plant part NR in intervention description	100 mL beverage containing 0.321 mg/mL red ginseng extract	Immediately after alcohol	Blood alcohol/acetaldehyde: 0, 30, 45, 60, 90, 120, 180, and 240 min; expiratory alcohol: 0–240 min at serial intervals; hangover symptom survey: 24 h
P06 Bang et al., 2015 (Korea)	Randomized, placebo-controlled, double-blind crossover	28; 28 M; 24.6 ± 1.6 years	Pure alcohol 20%, 1.75 g/kg over 2 h	Polysaccharide-rich extract of reported *Acanthopanax senticosus* root (current *Eleutherococcus senticosus*)	One bottle 30 min before + one bottle immediately after alcohol; extract amount NR	Pre + post alcohol	BAC: Serially for 3 h after completion of alcohol consumption; AHS, vital signs, and blood tests: The following morning
P07 Kim et al., 2017 (Korea)	Randomized controlled crossover	26; 26 M; 23.7 ± 0.3 years; heterozygous ALDH2	Korean soju 17.5%, 360 mL (∼50 g alcohol)	Standardized *Hovenia dulcis* Thunb. Fruit extract (HDE)	2460 mg HDE (6 capsules)	30 min before alcohol	Blood: Baseline and 1, 4, and 12 h post-treatment; hangover questionnaire: 1, 4, and 12 h post-treatment (alcohol consumed 30 min after study product)
P08 Mammen et al., 2018 (India)	Randomized, double-blind, placebo-controlled crossover	16; 16 M; 29.46 ± 6.24 years	Brandy 42.8%, 240 mL (∼1 g/kg/day stated)	Clovinol®; standardized *Syzygium aromaticum* clove-bud polyphenol extract	250 mg single capsule	Before alcohol ingestion	Blood/biochemical measures: 0, 0.5, 2, 4, and 12 h after treatment; hangover questionnaire: 14 h after alcohol consumption (after at least 7 h sleep)
P09 George et al., 2019 (Malaysia/USA)	Randomized, double-blind, placebo-controlled crossover	16 screened; 15 randomized/analyzed; 10 M/5 F; 25 ± 8.9 years	Jack Daniel’s whisky 40%; titrated to target BAL 0.12%	PHYLLPRO™; *Phyllanthus amarus* leaves	375 mg tablet twice daily for 10 days	Chronic pre-supplementation before challenge	BAL: Baseline, 0.5, 1, 1.5, 12, 18, and 22 h; HSS/POMS: Baseline and 2, 10, 12, 18, and 22 h; cytokine/liver panels: Baseline and 2, 12, and 22 h (times referenced to initiation of alcohol consumption)
P10 Hu et al., 2020 (Italy)	Open pilot registry; nonrandomized comparative groups	41; all M; group mean ages 25.4 ± 2.0 and 25.6 ± 2.4 years	Preceding alcohol exposure causing established hangover; amount/type NR	Robuvit® standardized oak wood extract; species NR in intervention description	300 mg/day for 3 days	Post-hangover/treatment setting	Baseline/inclusion and 24 h; the article states that all measurements were made 24 h after inclusion, with follow-up for 3 days
P11 Kim et al., 2023 Noni (Korea)	Randomized, double-blind, placebo-controlled crossover	30 analyzed; 30 M; 30.2 ± 1.2 years; participant-flow text internally inconsistent	Soju 20.1%, 360 mL (∼57 g alcohol)	*Morinda citrifolia* L. fruit extract	1 g Noni fruit extract/pack	30 min before alcohol	Hangover questionnaire: 60, 240, and 720 min; blood ethanol/acetaldehyde: 0, 20, 40, 60, 120, 240, and 720 min; aminotransferase safety endpoint: 720 min
P12 Jung et al., 2023 (Korea)	Randomized, double-blind, parallel, placebo-controlled	80 randomized; 76 analyzed; 45 M/31 F; 28.84 ± 5.13 years	Whisky 40%, 0.9 g/kg	HY_IPA: *Mesembryanthemum crystallinum* + *Pueraria lobata* flower + *Artemisia indica*	700 mg plant extract mixture in 80 mL beverage	30 min before alcohol	AHS: 12 h; blood ethanol/acetaldehyde: 0, 0.25, 0.5, 1, 2, 4, 6, and 13 h; blood ADH/ALDH activity: 0, 1, 2, 4, and 6 h
P13 Lee et al., 2024 (Korea)	Randomized, double-blind, placebo-controlled crossover	30 randomized; 25 analyzed; 14 M/11 F; group mean ages 27.0–31.0 years	Whisky 40%, 0.9 g/kg	HDPB/HDGB: *Hovenia dulcis* fruit + *Pueraria lobata* root or glutathione yeast; additional minor botanicals	500 mL beverage: HD 0.731% + PL 0.1% or GY 0.02%	30 min before alcohol	Hangover questionnaire: Approximately 15 h (next morning); blood alcohol/acetaldehyde: 0, 0.25, 0.5, 1, 2, 4, 6, and 15 h; safety/vital-sign assessments: 15 h
P14 Paik et al., 2024 (Korea)	Randomized, double-blind, placebo-controlled crossover	30 randomized; 24 analyzed; 12 M/12 F; group mean ages 26.33–32.80 years	Whisky 40%, 0.9 g/kg	HDB/HDPB/HDGB: *Hovenia dulcis* fruit ± *Pueraria lobata* root or glutathione yeast	500 mL beverage: HD 0.475% ± PL 0.1% or GY 0.02%	30 min before alcohol	Hangover questionnaire: 15 h (next morning); blood alcohol/acetaldehyde: 0, 0.25, 0.5, 1, 2, 4, 6, and 15 h; safety/vital-sign assessments: 15 h
P15 Hong et al., 2025 (Korea)	Randomized, double-blind, placebo-controlled crossover	40 randomized; 37 PP; baseline 28 M/12 F and age 25.23 ± 3.89 years reported for 40; completer sex NR	Soju 20.1%, 0.8 g/kg	FHR-SM: reported *Siegesbeckia glabrescens* + Mori ramulus (*Morus alba* twigs), 1:3	Test-product dose NR in primary article text available; GMP combined extract	30 min before alcohol	Blood alcohol/acetaldehyde PK: −30, 0, 7, 10, 15, 30, 60, 120, 240, and 900 min; AHSS: 240 min; AHS: 900 min; plasma SOD: −30, 120, and 240 min

**TABLE 2:** Characteristics of included animal studies.

Study	Design/sample	Species	Alcohol exposure	Botanical intervention/dose/route	Relevance to hangover	Outcome domains	Outcome assessment time point(s)
P11 Kim et al., 2023 Noni	Controlled rat experiment; n = 10/group	Male Sprague Dawley rats	40% ethanol, 3 g/kg oral	*Morinda citrifolia* fruit extract; 50 or 100 mg/kg; oral, 30 min pre-ethanol	Metabolic/PK model; no validated behavioral hangover surrogate	Ethanol/acetaldehyde PK; hepatic ADH/ALDH; liver enzymes	Serum ethanol/acetaldehyde: 1, 3, and 5 h; terminal liver tissue (used for ADH/ALDH and related analyses) collected after the 5 h blood sample
P16 Lee et al., 2014 ginseng berry	Controlled BALB/c mouse experiment	Male BALB/c mice; n = 20 total reported	20% ethanol, 10 mL/kg	Ginsenoside-free molecules from steam-dried *Panax ginseng* berries; 10 or 25 mg/kg pre-ethanol	Metabolic/mechanistic model; no behavioral hangover outcome	Serum ethanol/acetaldehyde; CYP2E1/catalase/ALDH expression	Blood and *in vivo* metabolic endpoints: 1 h after ethanol administration
P17 Lee et al., 2012 Korean pear	Controlled genotype/PK mouse experiments	Male Aldh2 +/+ and Aldh2^−/−^ mice; 64 total; 20.58–23.45 g	Ethanol gavage: 1 g/kg (ethanol PK) or 2 g/kg (acetaldehyde)	*Pyrus pyrifolia* juice/extract; 10 mL/kg before alcohol	PK/genotype model; no behavioral hangover outcome	Blood ethanol/acetaldehyde PK; ADH/ALDH; genotype interaction	Blood ethanol: 0, 0.25, 0.5, 1, 2, and 3 h; acetaldehyde was analyzed at its post-alcohol peak rather than as a full serial PK profile
P18 Je et al., 2021a sprouted ginseng (online 2020)	Controlled ICR mouse experiments; n = 7/group in main alcohol model	Male ICR mice	Ethanol 1.5 or 4.5 g/kg, intraperitoneal	Sprouted *Panax ginseng* (SG) 10 mL/kg; fermented SG 2.5 or 10 mL/kg; oral pre-ethanol	Includes righting-reflex recovery; preclinical behavioral endpoint only	Righting-reflex score/AUC; ethanol/acetaldehyde; ADH/ALDH; ALT/AST; oxidative/inflammatory endpoints	Righting-reflex behavior: 0–80 min after 4.5 g/kg ethanol; metabolic/liver endpoints: 1, 3, and 6 h; blood acetaldehyde and hepatic ADH/ALDH specifically reported at 1 and 3 h
P19 Je et al., 2021 Hovenia leaves/branches	Controlled male ICR mouse experiment; n = 5–8/group	Male ICR mice, 23–25 g	Ethanol 30%; 4.5 g/kg or 1.5 g/kg	*Hovenia dulcis* oxidized leaf/branch extract OLB (2:1), 0.2 g/kg; oral pre-ethanol	Includes righting-reflex recovery; preclinical behavioral endpoint only	Righting reflex; ethanol/acetaldehyde; ADH/ALDH; ALT/AST; redox/inflammation	Righting-reflex behavior: 0–80 min after 4.5 g/kg ethanol; ethanol/acetaldehyde and hepatic ADH/ALDH: 1 and 3 h; liver injury/redox/inflammatory endpoints: 3 h
P20 Kim et al., 2009 Oenanthe	Rabbit + ICR mouse controlled experiments	New Zealand white rabbits (∼2.5 kg) and male ICR mice (∼30 g)	Rabbit: 1.25 mL 20% ethanol IV; mouse: 240 µL 30% ethanol oral	*Oenanthe javanica* hot-water extract/fractions; rabbit 10% extract; mouse fractions 20–200 mg/kg	Metabolic/PK model; no behavioral hangover outcome	Plasma ethanol and acetaldehyde elimination; fraction-response	Rabbit plasma ethanol: Serial time course through 5 h after IV ethanol; mouse plasma ethanol/acetaldehyde: 2 h after treatment/alcohol administration
P21 Cho et al., 2005 Evodiae	Controlled ethanol-loaded ICR mouse experiment; n = 15/group	Male ICR mice, 20–25 g	Ethanol 20% or 40%; alcohol dose NR	Evodiae fructus (current *Tetradium ruticarpum*) methanolic/fraction extract; 2.5, 5, 10 mL/kg; co-administered	Mechanistic model; no behavioral hangover outcome	Plasma alcohol; hepatic ADH/ALDH and antioxidant gene expression	Plasma alcohol and hepatic ADH/ALDH/antioxidant-gene expression: 1, 2, 3, and 4 h after acute alcohol consumption

**TABLE 3:** Summary of evidence levels and key outcomes of included studies.

Study	Evidence level	Primary/direct outcome role	Secondary/supportive outcomes	Direction/statistical pattern	Safety reporting
P01 Pittler et al., 2003	L1	Primary: Overall hangover symptom score/20-item VAS difference	Secondary: POMS, cognitive battery, recovery time; safety	Null; primary and secondary efficacy comparisons not significant	Actively assessed; one facial-redness AE with artichoke
P02 Wiese et al., 2004	L1 + L2	Primary: 9-Item symptom index	Secondary: Severe-hangover incidence, wellbeing, CRP, vitals/labs	Mixed: Overall symptom index NS (P = 0.07); nausea, dry mouth, anorexia and severe-hangover incidence favored OFI	Vitals/labs assessed; AE reporting NR
P03 Takahashi et al., 2010	L1	Outcome hierarchy not prespecified; 22-item VAS/overall wellbeing	Symptom-resolution time; test-specific dosing comparisons	Mixed: Selected GI symptoms/wellbeing improved; complete symptom-resolution time NS	Formal AE monitoring NR
P04 Lee et al., 2013	L1 + L2	Role not explicitly designated; 14-item total/average hangover severity	PK ethanol/acetaldehyde; ALDH2-stratified symptom/PK findings	Positive direct symptom signal; genotype and PK effects heterogeneous	Clinical/lab safety parameters collected; AE details NR
P05 Lee et al., 2014	L1 + L2	Role not explicitly designated; 15-item symptom index	Plasma/expiratory alcohol, acetaldehyde, AUC; lab safety	Positive selected symptom signal; PK mixed, including higher acetaldehyde at a selected time	Vitals/labs assessed; AE details NR
P06 Bang et al., 2015	L1 + L2	Role not explicitly designated; acute hangover scale total/items	BAC/AUC/Cmax/Tmax; CRP, glucose, liver/renal labs	Positive symptoms; alcohol PK largely null; selected biomarkers changed	Vitals/labs assessed
P07 Kim et al., 2017	L1 + L2	Role not explicitly designated; validated 14-item questionnaire/total score	IL-6/IL-10, AST, alcohol/acetaldehyde; CYP2E1/ALDH2 modifiers	Positive direct symptom signal; biomarker/genotype associations	Safety/AE reporting NR
P08 Mammen et al., 2018	L1 + L2	Role not explicitly designated; 15-item hangover severity scale	Alcohol/acetaldehyde PK; CRP/IL-6; SOD/GSH/MDA/8-isoprostane	Positive symptom + biomarker signal	Two participants reported mild constipation
P09 George et al., 2019	L1 + L2	Primary: CRP/cytokines, liver tests, GSH-Px; secondary: HSS/POMS	Breath alcohol; tertiary safety labs/ECG/AE monitoring	Mixed: Overall HSS not significantly different; selected symptom/POMS and biomarker/PK findings occurred at specific time points	Actively assessed (CBC/metabolic panel/urinalysis/ECG/AEs)
P10 Hu et al., 2020	L1 + L2	Exploratory VAS symptom domains; no randomized primary endpoint	Oxidative-stress measure and routine labs	Positive (exploratory)	Paper states no side effects; monitoring method not clearly detailed
P11 Kim et al., 2023	L1 + L2 + L3	Human: Role not explicitly designated; 14-item symptom questionnaire	Human PK; linked rat PK/ADH/ALDH and liver markers	Mixed/mostly null human symptoms; selected symptom/subgroup findings; preclinical metabolic signal	Human AE reporting NR
P12 Jung et al., 2023	L1 + L2	Primary: Acute hangover scale total/items	Secondary: Alcohol/acetaldehyde PK, ADH/ALDH; safety	Positive: AHS total and items favored HY_IPA	Actively assessed; no severe AEs and no between-group AE difference reported
P13 Lee et al., 2024	L1 + L2	Role not explicitly designated; AHS total/items	Alcohol/acetaldehyde PK; ADH/ALDH assays; safety	Mixed: Total AHS NS (P = 0.3241); GI disorder significant	Actively assessed; no serious safety concern reported; total nonserious AE count unclear
P14 Paik et al., 2024	L1 + L2	Role not explicitly designated; AHS total/items	Alcohol/acetaldehyde PK; ADH/ALDH assays; safety	Null: Total and individual AHS differences NS	Actively assessed; no serious AEs; total nonserious AE incidence unclear
P15 Hong et al., 2025	L1 + L2	Primary: Alcohol/acetaldehyde PK	Secondary: AHSS/AHS symptoms and SOD	Mixed: Total AHSS/AHS NS; selected symptom items significant	Actively assessed; no serious AEs/discontinuations due AEs; article states no AEs observed
P16 Lee et al., 2014	L3	Preclinical metabolic endpoints	Serum ethanol/acetaldehyde; CYP2E1/catalase/ALDH expression	Preclinical directional signal	NR
P17 Lee et al., 2012	L3	Preclinical PK/genotype endpoints	Ethanol/acetaldehyde PK; ADH/ALDH; ALDH2 genotype	Mixed preclinical pattern (ethanol reduced; acetaldehyde increased in Aldh2^−/−^)	NR
P18 Je et al., 2021a	L3	Righting-reflex behavior + alcohol metabolism	Ethanol/acetaldehyde; ADH/ALDH; liver/oxidative/inflammatory outcomes	Mixed preclinical pattern: FSG 10 mL/kg behavioral signal; SG not significant in that endpoint	NR
P19 Je et al., 2021b	L3	Righting-reflex behavior	PK; ADH/ALDH; liver, redox and inflammatory endpoints	Preclinical directional signal across behavior and several biomarkers	NR
P20 Kim et al., 2009	L3	Plasma ethanol/acetaldehyde elimination	Extract/fraction comparisons and dose response	Mixed by fraction/dose; n-butanol fraction strongest	NR
P21 Cho et al., 2005	L3	Plasma alcohol concentration	ADH/ALDH and antioxidant gene expression	Mixed preclinical mechanistic pattern	NR

NR, not reported; NS, not significant. Outcome-assessment times are reported relative to alcohol exposure/intake unless the primary publication specified a different anchor. Detailed botanical identity, plant-part, extraction, and standardization information is provided in Supplementary Tables S2, S3.

Evidence levels: L1 = direct human hangover symptom outcomes; L2 = human pharmacokinetic, biochemical/mechanistic; L3 = preclinical animal evidence. Safety is reported separately.

In Table 3, Positive/Mixed/Null summarizes the pattern of direct human symptom findings where applicable and is descriptive rather than a quality or certainty rating.

The two 2024 Hovenia trials were treated as independent studies. Lee et al. (2024) registered their trial as KCT0009900 with ethics approval GIRB-24216-ZM, whereas Paik et al. (2024) registered their trial as KCT0009899 with ethics approval GIRB-24216-ZL. Despite similarities in site, period, and intervention family, the distinct registration and ethics identifiers support separate trial identities.

### 3.2 Human study characteristics

The 15 human studies comprised 13 randomized controlled/crossover trials with varying control and blinding procedures, one exploratory repeated self-controlled study, and one open non-randomized registry study (Table 1). Across these studies, 470 participants were enrolled or randomized and 437 were included in the reported analyses. Sample sizes ranged from 10 to 80 enrolled participants. Representative images of selected botanicals corresponding to included interventions are shown in Figure 2; these images are illustrative and do not represent study voucher specimens or exact intervention batches.

**FIGURE 2:**
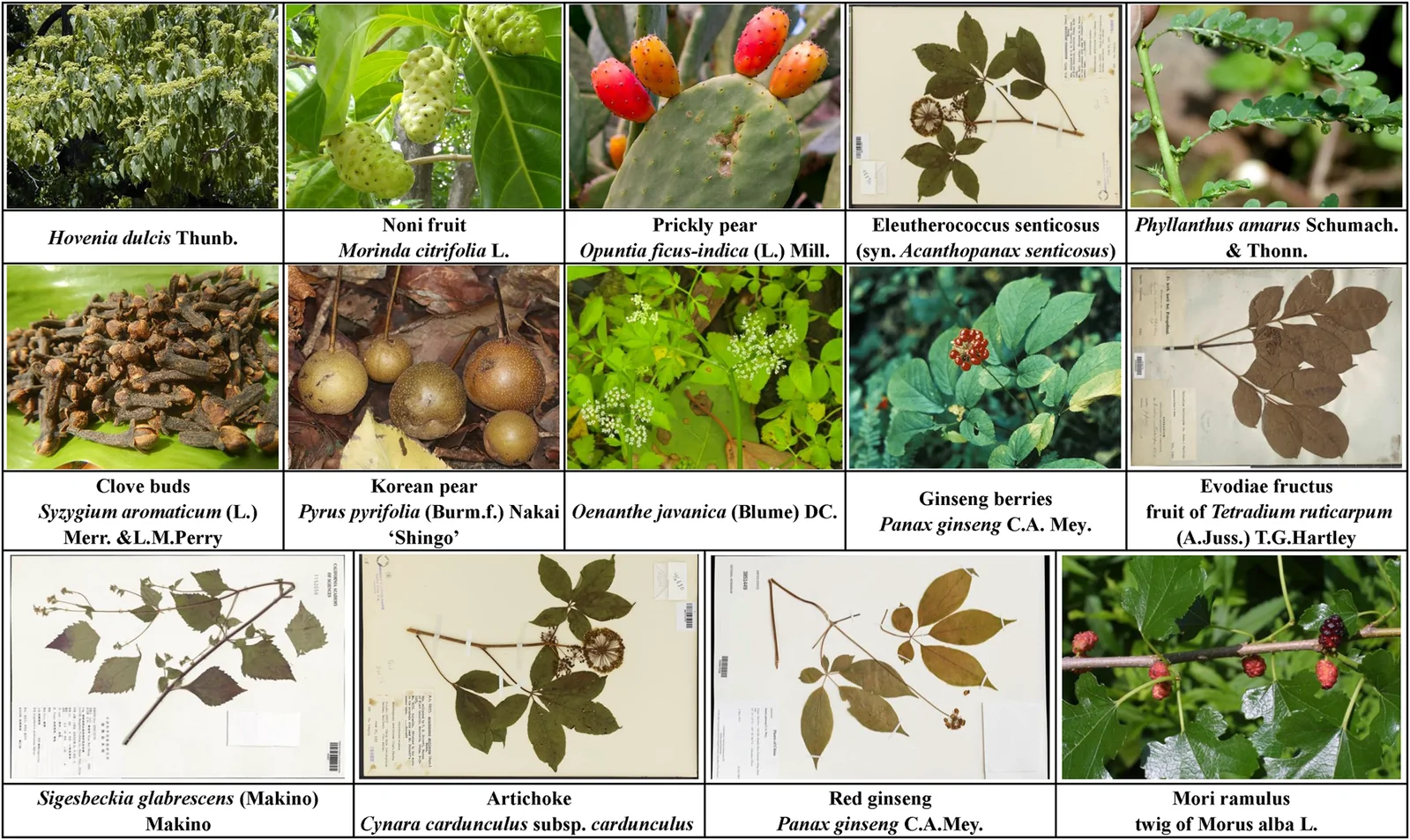
Representative images of botanicals corresponding to included interventions.

Sex distribution was available for the analyzed or completer cohort in 14 studies (n = 400), among whom 293 participants (73.3%) were male. In the remaining trial, 28 of 40 randomized participants were male, but sex distribution was not re-reported for the 37 completers; that study was therefore excluded from the analyzed-cohort sex denominator. The evidence base consequently remains substantially male-predominant and is concentrated in generally young, healthy adults.

Most interventions were administered before alcohol exposure and therefore addressed prevention rather than treatment of established hangover. Red ginseng and Robuvit were clearly administered after drinking or in participants with established hangover, whereas artichoke, KSS, and Acanthopanax protocols included mixed pre-/post-alcohol schedules (Pittler et al., 2003; Takahashi et al., 2010; Lee et al., 2014; Bang et al., 2015; Hu et al., 2020). This timing heterogeneity limits direct comparison between interventions intended to prevent symptoms and those intended to treat symptoms after they develop.

### 3.3 Level 1: direct human hangover symptom evidence

Direct symptom findings were heterogeneous. Seven publications were mapped as positive, six as mixed, and two as null. This direction map summarizes direct symptom outcomes only and does not incorporate biomarker or animal findings.

The two null reports were the artichoke trial and the 2024 Paik Hovenia trial. Pittler et al. (2003) found no significant difference in the prespecified 20-symptom visual-analogue-scale outcome or secondary efficacy outcomes. Paik et al. (2024) reported no significant between-treatment difference in total or individual Acute Hangover Scale (AHS) outcomes.

Mixed evidence included the *Opuntia ficus-indica* trial, in which the overall nine-item symptom index did not reach statistical significance (P = 0.07), although selected symptoms and the incidence of severe hangover favored the intervention (Wiese et al., 2004). The exploratory KSS study reported improvement in selected gastrointestinal symptoms and wellbeing, but time to complete symptom resolution was not significantly different (Takahashi et al., 2010). In the *Phyllanthus amarus* trial, overall Hangover Severity Score did not differ significantly between groups, although selected symptoms and Profile of Mood States domains differed at specific time points; symptom outcomes were secondary to biochemical/pharmacokinetic endpoints (George et al., 2019).

The Noni human trial was also mixed: total and most symptoms were not significantly different, dizziness at 240 min favored placebo (P = 0.031), whereas diarrhea at 720 min favored Noni (P = 0.036) (Kim et al., 2023). Lee et al. (2024) reported no difference in total AHS (P = 0.3241), although the gastrointestinal-disorder item differed (P = 0.0152). In the FHR-SM trial, total AHSS (P = 0.152) and AHS (P = 0.300) were nonsignificant, whereas selected symptom items differed; symptom scales were secondary to primary pharmacokinetic outcomes (Hong et al., 2026).

Positive direct symptom signals were reported for Korean pear, red ginseng, *Acanthopanax senticosus* extract, *Hovenia dulcis* fruit extract, clove-bud polyphenols, the exploratory Robuvit registry, and the HY_IPA plant-based mixture (Lee et al., 2013; Lee et al., 2014; Bang et al., 2015; Kim et al., 2017; Mammen et al., 2018; Hu et al., 2020; Jung et al., 2023). The strength of interpretation varies by design. In particular, the Robuvit study lacked randomization and placebo blinding (Hu et al., 2020). Jung et al. (2023) reported a prespecified AHS total score of 5.24 ± 5.78 versus 18.54 ± 18.50 (P < 0.0001) for HY_IPA versus placebo. Across the evidence base, statistically significant individual symptoms were not treated as establishing an overall intervention effect when the total or prespecified symptom outcome was nonsignificant.

### 3.4 Level 2: pharmacokinetic, biochemical, and pharmacogenetic outcomes

Human studies frequently measured blood or breath ethanol, acetaldehyde, AUC, Cmax, Tmax, inflammatory markers, oxidative-stress measures, liver-related laboratory tests, and ADH/ALDH-related biomarkers (Wiese et al., 2004; Lee et al., 2013; Lee et al., 2014; Bang et al., 2015; Kim et al., 2017; Mammen et al., 2018; George et al., 2019; Hu et al., 2020; Kim et al., 2023; Jung et al., 2023; Lee et al., 2024; Paik et al., 2024; Hong et al., 2026). These outcomes were analyzed separately from direct symptoms. Several trials showed nonuniform relationships between biomarker and symptom results. For example, red ginseng was associated with improvement in selected symptoms while plasma acetaldehyde was higher at a selected time point (Lee et al., 2014), and Acanthopanax produced a symptom signal without a material difference in blood-alcohol pharmacokinetics (Bang et al., 2015). The Noni, Lee 2024, Paik et al., 2024, and Hong studies likewise showed mixed pharmacokinetic findings that did not map consistently onto total symptom scores (Kim et al., 2023; Lee et al., 2024; Paik et al., 2024; Hong et al., 2026).

Genotype-related analyses were reported in the Korean pear and Hovenia studies (Lee et al., 2013; Kim et al., 2017). These findings were treated as pharmacogenetic or effect-modifier observations rather than evidence of genotype-dependent clinical efficacy unless a symptom-by-genotype interaction was directly demonstrated. Similarly, ADH, ALDH, and CYP2E1 activity or expression were classified as biochemical/mechanistic outcomes rather than pharmacokinetic endpoints.

Safety reporting was heterogeneous. Seven of the 15 human reports described active safety monitoring, one stated that no side effects were observed without clearly describing the monitoring method, and seven did not provide an explicit adverse-event result. Reported events included one facial-redness event with artichoke (Pittler et al., 2003), two mild constipation events with clove extract (Mammen et al., 2018), and mild events in the Phyllanthus study that were judged unrelated or unlikely related to the active product (George et al., 2019). The recent controlled trials reported safety monitoring without serious safety signals (Jung et al., 2023; Lee et al., 2024; Paik et al., 2024; Hong et al., 2026). These short-term observations do not establish long-term safety.

### 3.5 Level 3: preclinical animal evidence

Seven animal studies were retained as Level 3 evidence (Table 2). Five primarily evaluated alcohol-exposure metabolism, ethanol/acetaldehyde concentrations, enzyme activity/expression, or related biochemical outcomes without a behavioral hangover surrogate (Kim et al., 2023; Lee et al., 2014; Lee et al., 2012; Kim et al., 2009; Cho et al., 2005). Two studies evaluated loss/recovery of the righting reflex together with metabolic, liver, oxidative-stress, or inflammatory outcomes (Je et al., 2021a; Je et al., 2021b). Righting-reflex measures were interpreted as acute central nervous system sedation/recovery rather than as validated indicators of subjective human hangover. Accordingly, animal findings were used only to provide preclinical mechanistic context and not as evidence of clinical symptom efficacy.

### 3.6 Botanical identification and extract characterization

Reporting of botanical and extract characteristics was variable across the 21 included publications. Scientific botanical identity was reported in 19/21 (90.5%). Plant parts were fully reported in 16/21 (76.2%), partially reported in 3/21 (14.3%), and not reported in 2/21 (9.5%). Explicit botanical authentication was reported in 3/21 (14.3%), voucher numbers in 3/21 (14.3%), and a specific voucher depository in 2/21 (9.5%). Detailed taxonomic normalization, plant-part, material-source, authentication, and voucher information are provided in Supplementary Table S2.

Among the 19 publications for which an extraction solvent was applicable, the solvent was fully specified in 13 (68.4%), partially specified in two (10.5%), and not reported in four (21.1%). Extraction procedures were sufficiently described in 14/21 (66.7%) and partially described in 4/21 (19.0%). An author-reported DER or extraction yield was identified in 1/21 (4.8%); two additional values could be calculated from explicit source masses and were labelled as review-derived. Quantitative marker analysis or a defined phytochemical/chemical profile was reported in 8/21 (38.1%), whereas a batch or lot identifier was reported in 1/21 (4.8%). Detailed preparation, standardization, analytical-method, manufacturer, and batch information are provided in Supplementary Table S3.

## 4 Discussion

### 4.1 Principal findings

This scoping review identifies a substantially more heterogeneous clinical picture than a narrative focused only on positive findings would suggest. Direct human symptom evidence comprised positive, mixed, and null studies, and favorable changes in alcohol/acetaldehyde exposure or laboratory biomarkers did not consistently correspond to improvement in total hangover symptom scores. The central interpretive consequence is that direct symptoms, human biomarkers, and animal findings should not be combined into a single claim of “anti-hangover efficacy.”

The pattern is compatible with the caution raised by Roberts et al. (2022), whose systematic review of placebo-controlled randomized trials rated efficacy outcomes as very low quality because of methodological limitations and imprecision. The present scoping review does not perform an independent certainty assessment and therefore cannot upgrade or replace that conclusion. Its contribution is instead to map a broader and more recent evidence base, including mixed/null findings, newer trials published or first available online during 2023–2025, preclinical evidence, and botanical reporting characteristics.

### 4.2 Clinical heterogeneity and prevention versus treatment

The human literature remains small and heterogeneous in design, intervention, alcohol challenge, timing, and symptom measurement. Most studies administered the botanical preparation before alcohol exposure. These trials primarily address prevention, not treatment of an established hangover. Post-drinking treatment evidence was much less common. Future trials should define the clinical question prospectively and avoid pooling preventive and therapeutic timing as though they were interchangeable.

The descriptive positive/mixed/null map should also not be interpreted as a vote count for efficacy. A positive label may reflect a statistically supported direct symptom signal in a small or exploratory study, whereas a mixed label may reflect a nonsignificant total score with one or more significant individual symptoms. The artichoke and Paik trials demonstrate the importance of retaining null evidence (Pittler et al., 2003; Paik et al., 2024). Conversely, the strong AHS difference reported for HY_IPA warrants replication because a single trial cannot establish reproducible clinical benefit (Jung et al., 2023).

### 4.3 Pharmacokinetic and biochemical findings

Ethanol and acetaldehyde kinetics are biologically relevant to hangover, but their interpretation requires care. Acetaldehyde has pharmacological and toxic effects that can contribute to alcohol-related symptoms (Quertemont and Didone, 2006). Increased ADH activity may accelerate ethanol oxidation but could also increase acetaldehyde formation unless downstream clearance is adequate. Likewise, CYP2E1 participates in ethanol oxidation but is also linked to oxidative stress (Lu and Cederbaum, 2008). A lower ethanol concentration at one time point therefore does not by itself establish improved detoxification or symptom relief. Concentration-time profiles, acetaldehyde exposure, downstream metabolism, and direct symptoms should be considered together.

Inflammatory and oxidative-stress findings are similarly supportive rather than confirmatory. Alcohol consumption can trigger inflammatory responses that have been proposed to contribute to hangover symptomatology (van de Loo et al., 2020), while hydration status and water intake do not fully explain hangover severity (Mackus et al., 2024). Changes in CRP, cytokines, SOD, glutathione-related measures, or lipid-peroxidation markers can generate mechanistic hypotheses, but they do not establish mediation unless the study directly tests the relationship between biomarker change and symptom change.

### 4.4 Animal model validity

The animal literature is particularly vulnerable to overinterpretation. Animals cannot report headache, nausea, fatigue, thirst, mood change, or the overall subjective syndrome that defines human hangover. The retained animal studies therefore provide Level 3 evidence only. Righting-reflex loss or recovery is a measure of acute alcohol-induced central nervous system depression and recovery, not a validated hangover scale. Metabolic and organ-protective findings may be useful for hypothesis generation, but translation to human post-intoxication symptoms requires direct clinical testing.

### 4.5 Reproducibility of botanical interventions

The ConPhyMP-informed audit identified a second major limitation: incomplete characterization of the botanical preparations. Authentication and voucher documentation were uncommon, formal DER or yield information was rarely reported, and fewer than half of the publications provided quantitative marker analysis or a defined phytochemical profile. These reporting gaps do not prove that characterization was not performed; rather, they limit independent reproduction and meaningful comparison of the preparations.

Taxonomic normalization by review authors can resolve nomenclatural inconsistency but cannot retrospectively replace missing source authentication, plant-part documentation, extraction parameters, or chemical characterization. Future clinical and preclinical studies should prospectively follow ConPhyMP and the Four Pillars of Best Practice in Ethnopharmacology, report batch-specific characterization where relevant, and distinguish a named commercial product from the underlying botanical material and extract composition (Heinrich et al., 2022).

### 4.6 Safety, generalizability, and limitations

Safety evidence is insufficient for broad reassurance. Several studies actively monitored adverse events and laboratory parameters, but others provided no explicit adverse-event result, and follow-up was generally short. Absence of reported adverse events is not equivalent to demonstrated safety. Long-term use, interactions with medications, repeated use around heavy drinking, and safety in people with liver disease, older adults, or other comorbidities remain poorly characterized.

Generalizability is also limited. Among the analyzed participants for whom sex distribution was available, 73.3% were male, and most studies enrolled healthy younger adults. Samples were generally small, alcohol challenges and beverage types varied, and outcome instruments and assessment times were inconsistent. The two non-randomized/exploratory studies should not be interpreted as equivalent to blinded randomized trials. The review was restricted to English and Chinese publications and did not undertake formal risk-of-bias or certainty assessment, consistent with its scoping purpose; consequently, it cannot estimate comparative treatment effects or support clinical recommendations.

A further limitation concerns search chronology. The formal electronic database search ended on 22 July 2025. Five eligible reports were subsequently incorporated through citation searching and peer-reviewer recommendation and are shown separately as “other methods” in Figure 1 (the PRISMA flow diagram). This transparent separation avoids representing the supplementary identification process as a complete update of all eight databases.

### 4.7 Research priorities

Future research should prioritize adequately powered, prospectively registered randomized trials with validated total hangover symptom outcomes, prespecified primary endpoints, transparent multiplicity handling for individual symptoms, and clearly defined prevention or treatment timing. Pharmacokinetic and biochemical measures should be treated as secondary or mechanistic outcomes unless a study is specifically designed to test their relationship with symptoms. Trials should recruit more women and broader age and ethnic groups, report complete adverse-event data, and use standardized alcohol-challenge and follow-up procedures where ethically and clinically appropriate. For botanical interventions, reproducible taxonomic, manufacturing, extraction, and phytochemical documentation should be considered essential rather than optional.

## 5 Conclusion

The available literature on botanical interventions for alcohol hangover contains preliminary clinical signals but is limited by small samples, heterogeneous interventions and timing, mixed and null direct symptom findings, incomplete safety reporting, and variable botanical characterization. Human pharmacokinetic/biochemical findings and animal alcohol-exposure models provide supportive mechanistic context but should not be interpreted as direct evidence of symptom efficacy. On the current evidence, routine therapeutic use and long-term safety cannot be established. The principal value of this evidence map is to identify interventions and methodological gaps that warrant rigorous, reproducible clinical investigation.

## Data Availability

The original contributions presented in the study are included in the article/Supplementary Material, further inquiries can be directed to the corresponding authors.
