# Supplementary material for: Botanical interventions for alcohol hangover: a scoping review
Source: Front Pharmacol. 2026 Oct 26;17:1945900. doi: 10.3389/fphar.2026.1945900 (PMC13745484; doi:10.3389/fphar.2026.1945900)
Supplement: Supplementary file 2 [file Supplementaryfile2.docx]

Supplemental File 2: Search strategy for databases

Date(s): 2025 July 22^nd^

1. Pubmed Database

(((( "Alcohol Drinking/diet therapy"[Mesh] OR "Alcohol Drinking/drug therapy"[Mesh] OR "Alcohol Drinking/metabolism"[Mesh] OR "Alcoholic Intoxication/diet therapy"[Mesh] OR "Alcoholic Intoxication/drug therapy"[Mesh] OR "Alcoholic Intoxication/metabolism"[Mesh] )) OR ((((((((hangover*[Title/Abstract]) OR (veisalgia[Title/Abstract])) OR (acute alcoholism[Title/Abstract])) OR (Intoxication, Alcoholic[Title/Abstract])) OR (Drunkenness*[Title/Abstract])) OR (Drinking, Alcohol[Title/Abstract])) OR (Alcohol Consumption[Title/Abstract])) OR (Consumption, Alcohol[Title/Abstract]))) OR ((((("Alcohol Dehydrogenase/metabolism"[Mesh]) OR "Aldehyde Dehydrogenase/metabolism"[Mesh]) OR ( "Acetates/metabolism"[Mesh] OR "Acetates/poisoning"[Mesh] )) OR ( "Ethanol/metabolism"[Mesh] OR "Ethanol/poisoning"[Mesh] )) OR (((((((Alcohol-NAD+ Oxidoreductase[Title/Abstract])) OR (Alcohol Dehydrogenase II[Title/Abstract])) OR (D-Glucuronolactone Dehydrogenase[Title/Abstract])) OR (Aldehyde-NAD Oxidoreductase[Title/Abstract])) OR (Acetic Acid Esters[Title/Abstract])) OR (Acetic Acids[Title/Abstract])))) AND ((("Phytotherapy"[Mesh] OR "Drugs, Chinese Herbal"[Mesh] OR "Plant Extracts"[Mesh])) OR (((((Herb* Therapy[Title/Abstract]) OR (Extract*, Plant[Title/Abstract])) OR (Drugs, Plant[Title/Abstract])) OR ("Herbal Drugs"[Title/Abstract])) OR ("Plant Extracts"[Title/Abstract])))

712 records

2. Embase Database

#1 'drinking behavior'/exp

#2 'alcohol drinking':ti,ab OR 'controlled drinking':ti,ab OR 'drinking behaviour':ti,ab OR 'drinking habit':ti,ab OR 'drinking pattern':ti,ab OR 'social drinking':ti,ab OR 'drinking behavior':ti,ab

#3 'drug alcohol interaction'/exp

#4 'alcohol drug interaction':ti,ab OR 'drug ethanol interaction':ti,ab OR 'interaction, drug alcohol':ti,ab OR 'drug alcohol interaction':ti,ab

#5 'alcohol metabolism'/exp

#6 'ethanol metabolism':ti,ab OR 'alcohol metabolism':ti,ab

#7 'alcohol intoxication'/exp

#8 'alcohol intoxification':ti,ab OR 'alcoholic intoxication':ti,ab OR 'drunkenness':ti,ab OR 'ethanol intoxication':ti,ab OR 'inebriation':ti,ab OR 'intoxication, alcohol':ti,ab OR 'pathological drunkenness':ti,ab OR 'alcohol intoxication':ti,ab

#9 'hangover'/exp

#10 'hang-over':ti,ab OR 'hangover':ti,ab

#11 'veisalgia':ti,ab OR 'acute alcoholism':ti,ab OR 'intoxication, alcoholic':ti,ab OR 'drunkenness*':ti,ab OR 'drinking, alcohol':ti,ab OR 'alcohol consumption':ti,ab OR 'consumption, alcohol':ti,ab

#12 'alcohol dehydrogenase'/exp

#13 'alcohol nad oxidoreductase':ti,ab OR 'alcohol nicotinamide adenine dinucleotide oxidoreductase':ti,ab OR 'alcohol oxidoreductase':ti,ab OR 'alcohol oxidoreductases':ti,ab OR 'alcohol:nad oxidoreductase':ti,ab OR 'alcohol:nicotinamide adenine dinucleotide oxidoreductase':ti,ab OR 'alcoholdehydrogenase':ti,ab OR 'dehydrogenase, alcohol':ti,ab OR 'e.c. 1.1.1.1':ti,ab OR 'ethanol dehydrogenase':ti,ab OR 'nad and nadp dependent alcohol oxidoreductase':ti,ab OR 'nad and nadp dependent alcohol oxidoreductases':ti,ab OR 'nad (+) and nadp (+) dependent alcohol oxidoreductase':ti,ab OR 'nad (+) and nadp (+) dependent alcohol oxidoreductases':ti,ab OR 'alcohol dehydrogenase':ti,ab

#14 'aldehyde dehydrogenase'/exp

#15 'acetaldehyde dehydrogenase':ti,ab OR 'aldehyde dehydrogenase (nad)':ti,ab OR 'aldehyde nad oxidoreductase':ti,ab OR 'aldehyde nicotinamide adenine dinucleotide oxidoreductase':ti,ab OR 'aldehyde oxidoreductases':ti,ab OR 'aldehyde:nad oxidoreductase':ti,ab OR 'aldehyde:nad (p) oxidoreductase':ti,ab OR 'aldehyde:nicotinamide adenine dinucleotide oxidoreductase':ti,ab OR 'e.c. 1.2.1.3':ti,ab OR 'aldehyde dehydrogenase':ti,ab

#16 'd-glucuronolactone dehydrogenase':ti,ab OR 'acetic acid esters':ti,ab OR 'acetic acids':ti,ab

#17 'phytotherapy'/exp

#18 'medical plant therapy':ti,ab OR 'medicinal plant therapy':ti,ab OR 'phyto-medication':ti,ab OR 'phyto-therapy':ti,ab OR 'phytomedication':ti,ab OR 'plant therapy':ti,ab OR 'phytotherapy':ti,ab

#19 'herbaceous agent'/exp

#20 'drugs, chinese herbal':ti,ab OR 'herbaceous drug':ti,ab OR 'herbaceous plant':ti,ab OR 'herbaceous substance':ti,ab OR 'herbal agent':ti,ab OR 'herbal drug':ti,ab OR 'herbal material':ti,ab OR 'herbal medicinal product':ti,ab OR 'herbal preparation':ti,ab OR 'herbaceous agent':ti,ab

#21 'plant extract'/exp

#22 'plant extracts':ti,ab OR 'plant extract':ti,ab

#23 'herb* therapy':ti,ab OR 'extract*, plant':ti,ab OR 'drugs, plant':ti,ab OR 'herbal drugs':ti,ab

#24 #1 OR #2 OR #3 OR #4 OR #5 OR #6 OR #7 OR #8 OR #9 OR #10 OR #11 OR #12 OR #13 OR #14 OR #15 OR #16

#25 #17 OR #18 OR #19 OR #20 OR #21 OR #22 OR #23

#26 #24 AND #25

1087 records

3. Web of Science

#1 ((((((((((((((TS=(Alcohol Drinking/diet therapy)) OR TS=(Alcohol Drinking/drug therapy)) OR TS=(Alcohol Drinking/metabolism)) OR TS=(Alcoholic Intoxication/diet therapy)) OR TS=(Alcoholic Intoxication/drug therapy)) OR TS=(Alcoholic Intoxication/metabolism)) OR TS=(hangover*)) OR TS=(veisalgia)) OR TS=(acute alcoholism)) OR TS=(Intoxication, Alcoholic)) OR TS=(Drunkenness*)) OR TS= (Drinking, Alcohol)) OR TS=(Alcohol Consumption)) OR TS=(Consumption, Alcohol)) and Preprint Citation Index (Exclude – Database)

#2 ((((((((((((TS=(Alcohol Dehydrogenase/metabolism)) OR TS= (Aldehyde Dehydrogenase/metabolism)) OR TS= (Acetates/metabolism)) OR TS= (Acetates/poisoning)) OR TS= (Ethanol/metabolism)) OR TS= (Ethanol/poisoning)) OR TS= (Alcohol-NAD+ Oxidoreductase)) OR TS= (Alcohol Dehydrogenase II)) OR TS= (D-Glucuronolactone Dehydrogenase)) OR TS= (Aldehyde-NAD Oxidoreductase)) OR TS= (Acetic Acid Esters)) OR TS= (Acetic Acids)) and Preprint Citation Index (Exclude – Database)

#3 ((((((((TS=(Phytotherapy)) OR TS=(Herb* Therapy)) OR TS=(Herbal Drugs)) OR TS=(Plant Extracts)) OR TS=(Herb* Therapy)) OR TS=(Drugs, Chinese Herbal)) OR TS=(Extract*, Plant)) OR TS=(Drugs, Plant)) and Preprint Citation Index (Exclude – Database)

#4 #1 OR #2

#5 #3 AND #4

721 records

4. Cochrane Library Database

#1 MeSH descriptor: [Alcohol Drinking] explode all trees

#2 ("Alcohol Intake" OR "Intake, Alcohol" OR "Habits, Alcohol Drinking" OR "Drinking, Alcohol" OR "Alcohol Intakes" OR "Alcohol Drinking Habits" OR "Alcohol Drinking Habit" OR "Habit, Alcohol Drinking" OR "Alcohol Consumption" OR "Drinking Habit, Alcohol" OR "Consumption, Alcohol"):ti,ab

#3 MeSH descriptor: [Alcoholic Intoxication] explode all trees

#4 ("Intoxication, Alcoholic" OR "Drunkenness" OR "Drunkennesses"):ti,ab

#5 ("hangover" OR "veisalgia" OR "acute alcoholism" OR "Intoxication, Alcoholic" OR "Drunkenness" OR "Drinking, Alcohol" OR "Alcohol Consumption" OR "Consumption, Alcohol"):ti,ab

#6 MeSH descriptor: [Alcohol Dehydrogenase] explode all trees

#7 ("Alcohol Dehydrogenase II" OR "Alcohol-NAD+ Oxidoreductase" OR "Dehydrogenase, Alcohol" OR "Alcohol Dehydrogenase (NAD+)" OR "Oxidoreductase, Alcohol-NAD+" OR "Alcohol NAD+ Oxidoreductase" OR "Alcohol Dehydrogenase I" OR "Alcohol Dehydrogenase, Yeast" OR "Dehydrogenase, Yeast Alcohol" OR "Yeast Alcohol Dehydrogenase"):ti,ab

#8 MeSH descriptor: [Acetates] explode all trees

#9 ("Acetate" OR "Acetic Acid Esters" OR "Esters, Acetic Acid" OR "Acetic Acids" OR "Acids, Acetic"):ti,ab

#10 ("D-Glucuronolactone Dehydrogenase" OR "Aldehyde-NAD Oxidoreductase" OR "Alcohol Dehydrogenase/metabolism" OR "Aldehyde Dehydrogenase/metabolism" OR "Acetates/metabolism" OR "Acetates/poisoning" OR "Ethanol/metabolism" OR "Ethanol/poisoning"):ti,ab

#11 ("Alcohol Drinking/diet therapy" OR "Alcohol Drinking/drug therapy" OR "Alcohol Drinking/metabolism" OR "Alcoholic Intoxication/diet therapy" OR "Alcoholic Intoxication/drug therapy" OR "Alcoholic Intoxication/metabolism"):ti,ab

#12 MeSH descriptor: [Phytotherapy] explode all trees

#13 ("Herb Therapy" OR "Herbal Therapy"):ti,ab

#14 MeSH descriptor: [Drugs, Chinese Herbal] explode all trees

#15 ("Extracts, Chinese Plant" OR "Chinese Plant Extracts" OR "Plant Extracts, Chinese" OR "Chinese Herbal Drugs" OR "Herbal Drugs, Chinese" OR "Chinese Drugs, Plant"):ti,ab

#16 MeSH descriptor: [Plant Extracts] explode all trees

#17 ("Herbal Medicines" OR "Medicines, Herbal" OR "Extract, Plant" OR "Extracts, Plant" OR "Plant Extract"):ti,ab

#18 ("Herb Therapy" OR "Extract, Plant" OR "Drugs, Plant" OR "Herbal Drugs"):ti,ab

#19 #1 OR #2 OR #3 OR #4 OR #5 OR #6 OR #7 OR #8 OR #9 OR #10 OR #11

#20 #12 OR #13 OR #14 OR #15 OR #16 OR #17 OR #18

#21 #19 AND #20

158 records

5. CNKI

(SU='宿醉' OR SU='醉酒' OR SU='饮酒' OR SU='酒精中毒') AND (SU='中药' OR SU='草药' OR SU='中草药' OR SU='植物提取物' OR SU='天然植物')

163 records

6. Wanfang Data

主题:(("宿醉" or "醉酒" or "饮酒" or "酒精中毒") and ("中药" or "草药" or "中草药" or "植物提取物" or "天然植物"))

1430 records

7. VIP

(M="宿醉" OR M="醉酒" OR M="饮酒" OR M="酒精中毒") AND (M="中药" OR M="草药" OR M="中草药" OR M="植物提取物" OR M="天然植物")

113 records

8. CBM

("宿醉"[常用字段:智能] OR "醉酒"[常用字段:智能] OR "饮酒"[常用字段:智能] OR "酒精中毒"[常用字段:智能]) AND ("中药"[常用字段:智能] OR "草药"[常用字段:智能] OR "中草药"[常用字段:智能] OR "植物提取物"[常用字段:智能] OR "天然植物"[常用字段:智能])

841 records
