# Supplementary material for: Botanical interventions for alcohol hangover: a scoping review
Source: Front Pharmacol. 2026 Oct 26;17:1945900. doi: 10.3389/fphar.2026.1945900 (PMC13745484; doi:10.3389/fphar.2026.1945900)
Supplement: Supplementary file 3 [file Table1.docx]

Supplementary Table S1. Outcome-domain definitions and evidence levels.

| Outcome domain | Operational definition | Examples | Evidence level |
| --- | --- | --- | --- |
| Direct clinical hangover symptoms | Validated/structured human-reported hangover symptom scales, total scores, individual symptoms, overall well-being | AHS, AHSS, HSS, 14-/15-/20-/22-item questionnaires, VAS | L1 |
| Cognitive / mood outcomes | Human cognitive performance or mood/function measures assessed after alcohol exposure | POMS; memory/concentration; cognitive test batteries | Supportive human outcome |
| Pharmacokinetics / exposure | Measured ethanol/acetaldehyde exposure over time | BAC, breath alcohol, acetaldehyde, AUC, Cmax, Tmax, t1/2 | L2 human; L3 animal |
| Alcohol-metabolism enzymes | Biochemical activity/expression of enzymes involved in ethanol metabolism | ADH, ALDH, CYP2E1, catalase | L2 human; L3 animal |
| Inflammation | Inflammatory mediators or acute-phase responses | CRP, IL-6, IL-10, TNF-α, MCP-1 | L2/L3 |
| Oxidative stress / antioxidant defense | Redox biomarkers and antioxidant systems | SOD, GSH, catalase, MDA, 8-isoprostane, 4-HNE | L2/L3 |
| Organ function / protection | Hepatic, renal, structural or metabolic organ-injury measures | AST/ALT/GGT, creatinine/BUN, histology | L2 human; L3 animal |
| Neurobehavioral recovery | Animal behavior after alcohol exposure | LORR/righting-reflex latency, duration, behavioral score | L3 |
| Genotype modifier | Stratified symptom, PK or biomarker effects by genotype | ALDH2, CYP2E1 polymorphisms | L2 unless direct symptom-by-genotype effect |
| Safety | Adverse events and/or prespecified safety monitoring | AE interviews/questionnaires, vitals, CBC, chemistry, urinalysis, ECG | Reported separately |
| Outcome role | Author-designated position in the outcome hierarchy | Primary, secondary, tertiary/exploratory, role NR | Cross-cutting |
| Direction / significance | Descriptive synthesis of source-supported findings | Positive, Mixed, Null; significant/NS/descriptive | Cross-cutting |
