# Supplementary material for: Botanical interventions for alcohol hangover: a scoping review
Source: Front Pharmacol. 2026 Oct 26;17:1945900. doi: 10.3389/fphar.2026.1945900 (PMC13745484; doi:10.3389/fphar.2026.1945900)
Supplement: Supplementary file 4 [file Table2.docx]

Supplementary Table S2. Botanical identification and material-source characteristics.

| Study / intervention row | Name reported in primary article | Accepted botanical name (review-level) | Family | Plant part | Origin / source | Authentication | Voucher / depository |
| --- | --- | --- | --- | --- | --- | --- | --- |
| P01 Pittler 2003 | *Cynara scolymus* | *Cynara cardunculus* subsp. *cardunculus* | Asteraceae | NR | Commercial standardized extract | NR | NR |
| P02 Wiese 2004 | *Opuntia ficus indica* | *Opuntia ficus-indica* (L.) Mill. | Cactaceae | Fruit (dried cactus fruit; article also describes extract from skin of prickly pear fruit) | Commercial product Tex-OE, Extracts Plus Inc., San Diego, USA | NR | NR |
| P03 Takahashi 2010 | *Citrus tangerine* Hort. et Tanaka | *Citrus* × *aurantium* f. *deliciosa* (Ten.) M.Hiroe | Rutaceae | Pith | Ningxia Yinchuan Chinese Medicine Factory, China | NR | NR |
| P03 Takahashi 2010 | *Zingiber officinale* | *Zingiber officinale* Roscoe | Zingiberaceae | Rhizome | Uchida-Wakanyaku, Japan | NR | NR |
| P04 Lee 2013 | *Pyruspyrifolia* cv. Shingo (as reported) | *Pyrus pyrifolia* (Burm.f.) Nakai ‘Shingo’ | Rosaceae | Fruit | Korean pear source reported in sample-preparation methods | NR | NR |
| P05 Lee 2014 | NR (intervention described as red ginseng extract) | NR | NR | NR | NR | NR | NR |
| P06 Bang 2015 | *Acanthopanax senticosus* | *Eleutherococcus senticosus* (Rupr. & Maxim.) Maxim. | Araliaceae | Root (fresh roots) | Source of fresh roots not clearly identified in extracted section | NR | NR |
| P07 Kim 2017 | *Hovenia dulcis* Thunb. | *Hovenia dulcis* Thunb. | Rhamnaceae | Fruit | Lifetree Biotech, Suwon, Republic of Korea | Reported | D2015070441; article states a voucher was deposited |
| P08 Mammen 2018 | *Syzygium aromaticum* L. (as reported in article) | *Syzygium aromaticum* (L.) Merr. & L.M.Perry | Myrtaceae | Clove buds | Akay Flavours and Aromatics Ltd., Cochin, India | Identified/authenticated by botanist | AK-CLV-011; Herbarium of Akay Flavours and Aromatics Ltd., Cochin |
| P09 George 2019 | *Phyllanthus amarus* (article uses *P. amarus*; title gives species) | *Phyllanthus amarus* Schumach. & Thonn. | Phyllanthaceae | Leaves | Biotropics Malaysia Berhad | Identified by Shamsul Khamis, Institute of Bioscience, Universiti Putra Malaysia | SK3185/17 |
| P10 Hu 2020 | NR (standardized oak wood extract; Quercus robur appears in article background/reference context) | NR | NR | Wood | Commercial standardized product; article identifies Robuvit® | NR | NR |
| P11 Kim 2023 | *Morinda citrifolia* L. | *Morinda citrifolia* L. | Rubiaceae | Fruit | COSMAX NS, Seongnam-si, Gyeonggi-do, Republic of Korea | NR | NR |
| P12 Jung 2023 | *Mesembryanthemum crystallinum* | *Mesembryanthemum crystallinum* L. | Aizoaceae | NR | Materials supplied/used by hy Co., Ltd. study product | NR | NR |
| P12 Jung 2023 | *Pueraria lobata* flower | *Pueraria montana* var. *lobata* (Willd.) Maesen & S.M.Almeida ex Sanjappa & Predeep | Fabaceae | Flower | hy Co., Ltd. study product | NR | NR |
| P12 Jung 2023 | *Artemisia indica* | *Artemisia indica* Willd. | Asteraceae | NR | hy Co., Ltd. study product | NR | NR |
| P13 Lee 2024 | *Hovenia dulcis* Thunb. | *Hovenia dulcis* Thunb. | Rhamnaceae | Fruit | Concentrated aqueous sample provided by Kwangdong Pharmaceutical Co., Ltd., Gwacheon, Republic of Korea | NR | NR |
| P13 Lee 2024 | *Pueraria lobata* (root extract) | *Pueraria montana* var. *lobata* (Willd.) Maesen & S.M.Almeida ex Sanjappa & Predeep | Fabaceae | Root | Kwangdong Pharmaceutical Co., Ltd. | NR | NR |
| P13 Lee 2024 | *Acer tegmentosum* Maxim. (article table has spelling variant 'Maximowoc') | *Acer tegmentosum* Maxim. | Sapindaceae | NR | Kwangdong formulation | NR | NR |
| P13 Lee 2024 | *Alnus japonica* | *Alnus japonica* (Thunb.) Steud. | Betulaceae | NR | Kwangdong formulation | NR | NR |
| P14 Paik 2024 | *Hovenia dulcis* Thunb. | *Hovenia dulcis* Thunb. | Rhamnaceae | Fruit | Kwangdong Pharmaceutical Co., Ltd., Gwacheon, Republic of Korea | NR | NR |
| P14 Paik 2024 | *Pueraria lobata* root extract | *Pueraria montana* var. *lobata* (Willd.) Maesen & S.M.Almeida ex Sanjappa & Predeep | Fabaceae | Root | Kwangdong Pharmaceutical Co., Ltd. | NR | NR |
| P15 Hong 2025 | *Siegesbeckia glabrescens* Makino | *Sigesbeckia glabrescens* (Makino) Makino | Asteraceae | NR | GMP-manufactured FHR-SM; Frombio Co., Ltd., Yongin, Republic of Korea | NR | NR |
| P15 Hong 2025 | Mori ramulus; article describes twigs of *Morus alba* | *Morus alba* L. | Moraceae | Twig / ramulus | GMP-manufactured FHR-SM; Frombio Co., Ltd., Yongin, Republic of Korea | NR | NR |
| P16 Lee 2014 | *Panax ginseng* C.A. Meyer | *Panax ginseng* C.A.Mey. | Araliaceae | Berry / fruit | Fresh berries obtained from Korea Genetic Pharm (per source methods) | NR | NR |
| P17 Lee 2012 | *Pyrus pyrifolia* cv. Shingo | *Pyrus pyrifolia* (Burm.f.) Nakai ‘Shingo’ | Rosaceae | Fruit | Korean pear source reported in sample preparation | NR | NR |
| P18 Je 2021 | *Panax ginseng* | *Panax ginseng* C.A.Mey. | Araliaceae | Whole sprout / whole plant | Sprouted ginseng harvested after 25–30 days; source locality/supplier reported in methods | NR | NR |
| P19 Je 2021 | *Hovenia dulcis* | *Hovenia dulcis* Thunb. | Rhamnaceae | Leaves and branches | Malgeunsan Agricultural Union, Sanchung/Gyeongnam, Republic of Korea; harvested Oct–Nov 2021 | NR | NR |
| P20 Kim 2009 Oenanthe | *Oenanthe javanica* DC | *Oenanthe javanica* (Blume) DC. | Apiaceae | Aerial parts | Agricultural farms in Pyongyang-ri, Cheongdo-gun, Gyeongsangbuk-do, Republic of Korea | NR | NR |
| P21 Cho 2005 Evodiae | *Evodia rutaecarpa* / Evodiae fructus (as article terminology) | *Tetradium ruticarpum* (A.Juss.) T.G.Hartley | Rutaceae | Dried unripe fruit | Purchased from an herb market in Seoul, Republic of Korea | NR | Voucher specimens deposited at Herbarium of Natural Products Research Institute, Seoul National University (number NR) |
