# Supplementary material for: Botanical interventions for alcohol hangover: a scoping review
Source: Front Pharmacol. 2026 Oct 26;17:1945900. doi: 10.3389/fphar.2026.1945900 (PMC13745484; doi:10.3389/fphar.2026.1945900)
Supplement: Supplementary file 5 [file Table3.docx]

Supplementary Table S3. Extract preparation and phytochemical characterization.

| Study | Preparation / product | Extract / preparation type | Solvent | Extraction procedure | DER / yield | Standardization / markers | Analytical method / fingerprint | Manufacturer / batch |
| --- | --- | --- | --- | --- | --- | --- | --- | --- |
| P01 Pittler 2003 | LI120 artichoke | Commercial standardized extract | NR | NR | 1.92 g/day corresponds to ~48 g fresh artichoke; formal DER NR | Standardized commercial extract; specific marker NR | NR | Commercial product; manufacturer not identified in extracted text |
| P02 Wiese 2004 | Tex-OE / OFI | Dried fruit solvent extract | Standard solvent; identity/proportion NR | Cactus fruit dried then standard solvent extraction | NR | NR | NR | Extracts Plus Inc., San Diego, USA |
| P03 Takahashi 2010 | KSS formula | Aqueous decoction + granulation | Tap water; 70–80% edible ethanol used for granulation | 600 g Kitsuraku +1200 g Shokyo boiled in 15 L tap water 45 min; strained/lyophilized; extract blended with brown sugar/dextrin and granulated | 210 g lyophilized extract from 1800 g starting botanicals (~11.7% combined yield; DER ~8.57:1 calculated from reported masses, not stated by authors) | NR | NR | Kitsuraku: Ningxia Yinchuan Chinese Medicine Factory; Shokyo: Uchida-Wakanyaku |
| P04 Lee 2013 | Korean pear juice | Fresh juice | None / fresh juice | Fruit cut and processed with expeller juicer; filtered with Whatman No.2; vacuum packed | NA (fresh juice; no drug-to-extract ratio applicable) | NR | NR | Pear source reported in sample-preparation methods |
| P05 Lee 2014 | Red ginseng drink | Water extract beverage | Water | Article states red ginseng drink was made by water-extract method | NR | Beverage contained 0.321 mg/mL red ginseng extract; specific chemical marker/content NR | NR | Korean Ginseng Corporation, Korea |
| P06 Bang 2015 | PEA | Aqueous polysaccharide-rich extract | Water | Fresh roots washed/shade-dried/sliced; blanched 100°C 4 h at 43.3 g/L; chromatographic/polysaccharide enrichment; ethanol precipitation; freeze-dried | NR | Polysaccharide-rich; reported ~85% carbohydrate | Sephadex G-150 fractionation and carbohydrate characterization | Prepared for study; supplier/source of roots NR in extracted section |
| P07 Kim 2017 | HDE | Aqueous standardized extract | Distilled water | H. dulcis fruit boiled 4 h; filtered/concentrated; freeze-dried; mixed with dextrin 10:6 | Formal extraction yield/DER NR; dextrin mixing ratio 10:6 | Quercetin 5.9–8.9 mg/g | HPLC (Agilent 1200) | Lifetree Biotech, Suwon, Republic of Korea |
| P08 Mammen 2018 | Clovinol® | Hydroethanolic standardized clove-bud polyphenol extract | Hydroethanolic; ratio NR | Hydroethanolic extraction followed by purification, emulsification and spray drying | NR | Total polyphenols 28.6% gallic-acid equivalent; multiple characterized polyphenols | Folin–Ciocalteu + UPLC-QTOF characterization | Akay Flavours and Aromatics Ltd., Cochin, India; batch/lot: 01/13 B2 (July 2013) |
| P09 George 2019 | PHYLLPRO™ | Standardized hydroethanolic/ethanol-water extract | Ethanol:water 1:1 | Air-dried leaves ground; extracted 4 h at 45°C; filtered; evaporated 60–80°C; spray-dried | NR | ≥0.1% hypophyllanthin; ≥0.4% phyllanthin; total phenolics ≤100 mg GAE/g dry extract (as reported) | HPLC for lignans; total phenolics assay | Biotropics Malaysia Berhad; product manufactured under GMP by Unison Nutraceutical Sdn Bhd |
| P10 Hu 2020 | Robuvit® | Commercial standardized oak wood extract | NR | NR | NR | Standardized product; specific marker/content NR in extracted primary text | NR | Horphag Research is referenced in product literature/background; primary extracted intervention manufacturer should be treated cautiously |
| P11 Kim 2023 | Noni fruit extract | Hydroethanolic fruit extract | 10% ethanol; 10 volumes by weight | Dried/crushed fruit mixed with 10 volumes 10% ethanol; 80°C 4 h; filtered; reduced-pressure concentration at 70°C; sterilized; high-temperature instantaneous drying/cooling; 60-mesh sieve | NR | Polysaccharide/fractional chemical characterization reported; no single standardized marker clearly specified | Chromatographic/molecular-weight characterization described in article | COSMAX NS, Seongnam-si, Republic of Korea |
| P12 Jung 2023 | HY_IPA beverage | Plant-based extract mixture beverage | NR | Mixture incorporated into 80 mL beverage; detailed extraction procedures/ingredient ratios not reported in extracted text | NR | NR | NR | hy Co., Ltd. |
| P13 Lee 2024 | HDPB / HDGB beverage | Concentrated aqueous extracts in beverage | Water / aqueous concentrate | Concentrated aqueous HD (60 Brix; 50% solids) + PL (50 Brix; 40% solids) or glutathione yeast; beverage formulation provided | Formal DER/yield NR | GY is yeast extract 100% with L-glutathione >10%; botanical marker standardization NR | NR | Kwangdong Pharmaceutical Co., Ltd., Gwacheon, Republic of Korea |
| P14 Paik 2024 | HDB / HDPB / HDGB beverage | Concentrated aqueous extracts in beverage | Water / aqueous concentrate | Concentrated aqueous HD and PL/GY incorporated in 500 mL beverage | Formal DER/yield NR | GY >10% L-glutathione; botanical marker standardization NR | NR | Kwangdong Pharmaceutical Co., Ltd., Gwacheon, Republic of Korea |
| P15 Hong 2025 | FHR-SM | Combined commercial botanical extract | NR | GMP-manufactured combined extract; detailed solvent/process NR in extracted main text | NR | NR | NR | Frombio Co., Ltd., Yongin, Republic of Korea |
| P16 Lee 2014 | GFM fraction | Steam-dried berry ethanol extract / ethyl-acetate fraction | 80% aqueous ethanol; subsequent ethyl acetate fractionation | Fresh berries steamed 100°C 2 h, dried 50°C 24 h (repeated processing); 100 g material steeped in 1 L 80% ethanol 24 h repeatedly over 3 days; combined; ethyl-acetate fraction/column workup | Approximately 50% GFM dry yield from ethanol extracts (as reported for fraction) | Ginsenoside-free fraction | GC-MS chemical characterization | Korea Genetic Pharm (fresh berries); batch/lot NR |
| P17 Lee 2012 | Korean pear extract/juice | Fresh juice | None / fresh juice | Pears cut into equal pieces; expeller juicer; Whatman No.2 filtration; vacuum packed | NA (fresh juice; no drug-to-extract ratio applicable) | NR | NR | Source reported in sample preparation |
| P18 Je 2021 | SG / FSG | Aqueous extracts; fermentation/processing for FSG | Deionized water | SG: dried 55°C 3 d then ~60× DI water at 100°C 12 h; FSG: steam 100°C 30 min, age 75°C 10 d, dry 55°C 3 d, ~20× water 100°C 4 h; fermentation details reported in paper | NR | Phenolic/flavonoid enrichment characterized | Chemical-content assays in article | Source/locality reported in methods |
| P19 Je 2021 | OL / BE / OLB | Water extracts of oxidized leaves and branches | Water | 4 g leaf or branch +100 mL water, 95°C 3 h; filtered and freeze-dried; OLB combined 2:1 by freeze-dried extract weight | NR | Catechin, chlorogenic acid, taxifolin, ferulic acid, quercetin and other phenolics quantified/profiled | HPLC | Malgeunsan Agricultural Union, Sanchung/Gyeongnam, Republic of Korea |
| P20 Kim 2009 | Hot-water extract and fractions | Hot-water extract + solvent fractions | Boiling water; n-hexane, methylene chloride, n-butanol fractionation | 600 g dried aerial parts refluxed with 6 L boiling water 3 h; ~165–180 g residual hot-water extract; sequential liquid-liquid extraction | Hot-water yield ~27.5–30% w/w calculated from reported masses; authors report residual mass 165–180 g from 600 g | Fractions studied; individual marker standardization NR | NR | Collected from farms in Pyongyang-ri, Cheongdo-gun, Gyeongsangbuk-do, Republic of Korea |
| P21 Cho 2005 | Evodiae fructus fraction | Methanolic extract / organic-solvent fraction | 100% methanol; then n-hexane and ethyl acetate partitioning | 1 kg dried material sliced; extracted 3× with 10 L 100% MeOH at room temperature; diluted to 90% MeOH; partitioned/extracted with n-hexane/ethyl acetate; N2 evaporation | NR | NR | NR | Herb market, Seoul, Republic of Korea |
