# Supplementary material for: Botanical interventions for alcohol hangover: a scoping review
Source: Front Pharmacol. 2026 Oct 26;17:1945900. doi: 10.3389/fphar.2026.1945900 (PMC13745484; doi:10.3389/fphar.2026.1945900)
Supplement: Supplementary file 6 [file Table4.docx]

Supplementary Table S4. Full-text records excluded after eligibility reassessment.

| ID | Citation | Population | Principal exclusion reason | Final status |
| --- | --- | --- | --- | --- |
| X01 | Jung 2015 GABA black sticky rice | Human | Acute intoxication only / no eligible hangover symptom outcome | Exclude |
| X02 | Du 2010 Semen Hoveniae | Animal | Organ injury only / no eligible hangover-related outcome | Exclude |
| X03 | Xie 2022 MLG | Animal | Wrong condition: acute alcohol intoxication | Exclude |
| X04 | Shen 2012 DHM | Animal | Wrong condition: acute intoxication; isolated metabolite | Exclude |
| X05 | Kimura 2013 Curcuma zedoaria | Animal | Wrong condition: alcohol-induced drunkenness / acute intoxication | Exclude |
| X06 | Li 2024 Lingjiao Gouteng decoction | Animal | Wrong condition: acute alcohol intoxication | Exclude |
| X07 | Ramudu 2011 ginger | Animal | Wrong condition: chronic alcohol-related organ injury | Exclude |
| X08 | Chang 2013 Noni juice | Animal | Wrong condition: chronic alcohol-related hepatotoxicity | Exclude |
